# Towards Efficient Acidic Catalysts via Optimization of SO_3_H-Organosilane Immobilization on SBA-15 under Increased Pressure: Potential Applications in Gas and Liquid Phase Reactions

**DOI:** 10.3390/ma14237226

**Published:** 2021-11-26

**Authors:** Maciej Trejda, Ada Kaszuba, Ardian Nurwita, Maria Ziolek

**Affiliations:** Department of Heterogeneous Catalysis, Faculty of Chemistry, Adam Mickiewicz University, Uniwersytetu Poznańskiego 8, 61-614 Poznań, Poland; ada.jadrzak@wp.eu (A.K.); ardnur@st.amu.edu.pl (A.N.); ziolek@amu.edu.pl (M.Z.)

**Keywords:** ordered silica, modification with SO_3_H organosilanes, acetic acid esterification, glycerol dehydration

## Abstract

In this paper, the optimization of the synthesis of catalysts based on acidic mesoporous silica of the SBA-15 type by post-synthesis immobilization of 3-(trihydroxysilyl)-1-propanesulfonic acid (TPS) under increased pressure up to 20 bar is reported. Sample structures and composition were examined by XRD measurement, low-temperature N_2_ adsorption/desorption and elemental analysis. The catalytic activities of the materials obtained were determined in both gas and liquid phase processes, i.e., by esterification of acetic acid and glycerol dehydration, respectively. The optimum pressure for modification leading to the highest number of acidic sites was found to be 10 bar. The final material was very active and stable in liquid phase processes; however, the stability in the gas-phase process was unsatisfactory due to the loss of sulphonic species from the catalyst surface.

## 1. Introduction

Numerous chemical transformations involving acid catalysts are of great importance in industrial applications. This group includes a significant number of processes performed in the homogeneous phase, usually catalyzed by mineral acids [1]. A well-known example is the transesterification process leading to biofuel formation [2]. Although the yield of products that can be obtained in the homogenous phase is high, this method of production is charged with a series of drawbacks. First, the catalyst cannot be easily separated from the reaction mixture, which implies the formation of waste products as a result of the neutralization of mineral acid. This results in a greater burden on the natural environment and is against the rules of so-called green chemistry [3]. Other important disadvantages of the homogeneous processes are water pollution and the corrosion of the apparatus. Most of the aforementioned problems can be eliminated by application of solid catalysts having acidic sites on their surface.

The discovery of mesoporous silicas of different types, e.g., MCM-41 [4], SBA-15 [5], MCF [6], has inspired attempts to functionalize their surfaces, which can endow them with different properties. An important task in this regard is the generation of Brønsted acid sites. One of the first such attempts was based on incorporation of Al species into the structure of mesoporous silica to obtain a composition similar to that of zeolites [7]. Unfortunately, the generated acidity was much weaker when compared to that of zeolites. Later, different organosilane species were used as modifiers of mesoporous silicas. Usually, 3-mercaptopropyl)trimethoxysilane (MPTMS) was applied for this purpose, incorporated both during and after the synthesis of the support [8,9,10]. However, MPTMS contains SH species that have to be oxidized to sulfonic ones using, e.g., hydrogen peroxide. This process has to be performed in the course of a one-pot synthesis or in a separate step of post-synthesis modification. The acidity of the materials thus obtained is determined by both the number and strength of the generated acid sites. A high number of acidic sites can be achieved by a high efficiency of organosilane species immobilization, whereas the strength of SO_3_H species depends on the kind of functional groups linked to the hydrocarbon chain [11,12]. For instance, the presence of electrophilic groups in the neighborhood of SO_3_H species will make a proton easier to remove, increasing its acidity.

**Scheme 1:**
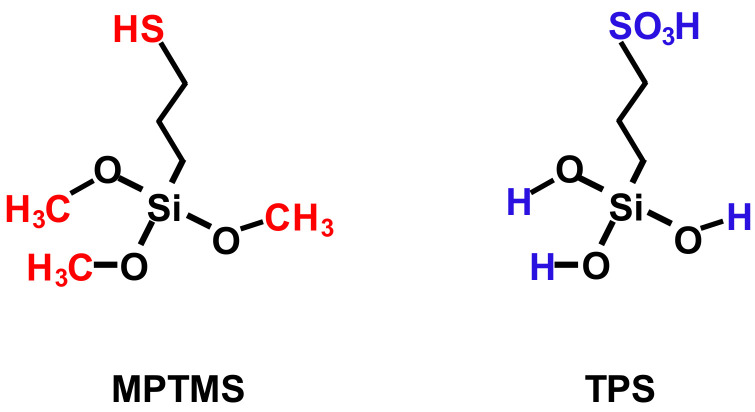
Structural formula of MPTMS and TPS.

Recently, we have succeeded in the post-synthesis modification of mesoporous materials of SBA-15 with MPTMS species using elevated pressure, which allowed us to increase the efficiency of organosilane species incorporation [13]. As an alternative, 3-(trihydroxysilyl)-1-propanesulfonic acid (TPS) species were also incorporated on SBA-15 during a one-pot synthesis approach, as well as by post-synthesis modification using microwave heating [14,15]. The structural formulae of both organosilane modifiers are presented in Scheme 1. The method involving the use of microwave radiation also allowed us to increase the number of acidic sites. It was established that the highest incorporation efficiency of organosilane species on SBA-15 was achieved under the maximum pressure applied in this study (8.5 bar). Therefore, the question arises whether a higher pressure would give even better results, and whether there is some optimal pressure for such a modification. Moreover, it should be pointed out that the immobilization of MPTMS on the silica surface occurs via the interaction of methoxy groups from MPTMS and silanol groups from the support. As a result of MPTMS immobilization, methanol molecules are formed. In the case of TPS species, two OH groups from the organosilane and silica surfaces participate in the immobilization process in which water molecules are released. Therefore, the question also arises whether the immobilization of TPS species is also sensitive to an increase in pressure during the modification process. In view of the above, the main goal of this study was to examine the impact of pressures up to 20 bar applied during post-synthesis modification of SBA-15 on the efficiency of TPS species incorporation. Evaluation of the catalytic activity of the materials prepared in this way was also an aim of this work.

## 2. Materials and Methods

### 2.1. Materials

All chemicals and materials used were purchased from commercially available sources and used without further purification. Pluronic P123, tetraethyl orthosilicate—TEOS (>99%), toluene (anhydrous), n-butanol (>99%), n-hexanol (>99%) and glycerol were purchased from Aldrich (St. Louis, MO, USA); HCl (35%) and acetic acid (99.5–99.9%) were procured from Stanlab (Lublin, Poland); 3-(trihydroxysiyl)-1-propanesufonic acid (30–35% in water) was purchased from Gelest (Morrisville, PA, USA).

### 2.2. Preparation of SBA-15 Support

SBA-15 material was obtained via one-pot hydrothermal synthesis, adapted from [5]. First a mixture of HCl (8.76 g) and water (141.24 g) was prepared. Then, 4 g Pluronic P123 (Poly(ethylene glycol)-block-poly(propylene glycol)-block-poly(ethylene glycol) was dissolved in the above solution. Then, 8.527 g TEOS was added dropwise to the solution. After stirring at 313 K for 20 h and heating at 373 K under static conditions for the next 24 h the product was filtered, washed with water (1000 mL) and dried at room temperature (r.t.). The template was removed by calcination at 823 K for 8 h (temperature ramp 5 K min^−^^1^).

### 2.3. Preparation of the TPS-Containing Catalysts

Immobilization of 3-(trihydroxysilyl)-1-propanesufonicacid(TPS) was performed in a 50 mL Parr reactor. For this purpose, 1 g of the support was immersed in 30 mL of toluene. Next, TPS was added (Si/TPS molar ratio = 10, estimated in [14]) and the reactor was closed. The pressure was set at 1, 5, 10, 15 or 20 bar by the addition of inert gas (helium). The modification was performed at 383 K for 24 h. Finally, the sample was filtrated, washed with 130 mL of toluene, 130 mL of ethanol and 130 mL of water, and dried at RT. Materials obtained were labeled as TPS/SBA-15-x, where x stands for the value of pressure during modification.

### 2.4. Preparation of Vanadium and Niobium Modified SBA-15

Vanadium and niobium were incorporated onto the surface of SBA-15 via incipient wetness impregnation. Prior to the modification, 1.5 g of the support was outgassed in an evaporator for 0.5 h at 353 K. Then, the support was flooded with a methanol solution of vanadium(V) oxytriisopropoxide or niobium(V) ethoxide in an amount which ensured that only the pores of SBA-15 would be filled. The amount of V or Nb in the solution was sufficient to obtain 5 wt.% of these metals in the final product. The mixture was rotated and heated in an evaporator at 313 K for 30 min. The powder obtained was filtered, dried at 333 K for 24 h and calcined at 773 K for 6 h in air in steady state conditions (heating rate 5 K min^−^^1^).

### 2.5. Characterization Techniques

Low temperature N_2_ adsorption/desorption measurements were performed using the Micromeritics ASAP 2020 instrument (Norcross, GA, USA). Prior to the analysis, the sample (ca. 100 mg) was outgassed at 373 K under vacuum (<1.3 Pa) for 20 h. The surface area of samples was calculated using the BET method, with a measurement error of less than 2 m^2^ g^−^^1^. Pore volume and diameter were determined by DFT method.

XRD measurements were performed using a Bruker AXS D8 Advance diffractometer (Bruker, Karlsruhe, Germany) with Cu Kα radiation (λ = 0.154 nm) at a step of 0.05° s^−^^1^.

Elemental analyses of the solids were carried out with Elementar Analyser Vario EL III (Elementar Analysensysteme GmbH, Hanau, Germany).

UV-VIS spectra were recorded using a Varian-Cary 300 Scan UV-Visible Spectrophotometer (Candela, Warszawa, Poland)

### 2.6. Esterification Process

Esterification of acetic acid with n-butanol or n-hexanol was performed in a liquid phase in a glass reactor placed in an EasyMax Work Station, using the catalyst having the highest number of acid sites. Prior to the reaction, the catalyst was activated in the air in a steady state condition at 423 K for 12 h (temperature ramp 5 K min^−^^1^). A mixture of acetic acid (40 mmol) and alcohol (80 mmol) was prepared, to which 100 mg of the catalyst was added. The reaction was performed for 4 h at 373 K (stirring rate—800 rpm). Qualitative analysis was conducted using a GC-MS chromatograph (Thermo Trace 1300) equipped with a DB-1 column (30 m) to determine possible formation of side products; no side products were detected. To determine product concentration, the residual acid was titrated using 0.1 M solution of NaOH. After the reaction, the catalysts were separated and dried at 383 K for 18 h. Catalyst stability was checked by its application in the following catalytic cycles.

### 2.7. Oxidative Dehydrogenation of Glycerol

The reaction was performed with the use of two catalyst beds. Portions of 0.05 g of granulated (0.5 < ø < 1 mm) TPS/SBA-15-10 or Nb/SBA-15 catalyst (top layer in the reactor) and of 0.05 g of granulated (0.5 < ø < 1 mm) V/SBA-15 material (bottom layer) were placed in the reactor and activated at 673 K for 2 h under argon flow (50 mL min^−^^1^). Then, the temperature was set to 623 K for the reaction. Next, 4 mL of aqueous solution of glycerol (10 wt.%) was passed continuously for 4 h using a pump system (KD Scientific) together with argon, used as a carrier gas (40 mL min^−^^1^), and oxygen (10 mL min^−^^1^). The reaction products were collected within each hour in an aqueous solution of hydroquinone (0.001 wt.%, 5 mL), then analyzed on a gas chromatograph (Thermo Scientific, Waltham, MA, USA) equipped with a DB-1 column (30 m) and an MS detector. Helium was used as a carrier gas. The concentrations of the reaction products were determined using the calibration curves.

## 3. Results and Discussion

In this study, SBA-15 was functionalized with 3-(trihydroxysilyl)-1-propanesulfonic acid (TPS) following its synthesis at different pressures (from 1 to 20 bar) during the modification process. The materials obtained were labelled as TPS/SBA-15-x, where x stands for the value of pressure applied during the immobilization of TPS species. The reference sample with acidic properties (Nb/SBA-15) was also prepared in order to compare the stabilities of the organosilane-containing materials in the gas-phase test reaction (oxidative dehydrogenation of glycerol). A V/SBA-15 sample was also prepared as a source of the active sites involved in the oxidation step of the above process. The activity and stability of the TPS/SBA-15-10 material in the liquid phase was checked using esterification of acetic acid with n-butanol and hexanol.

### 3.1. Textural/Structural Characterization

**Figure 1:**
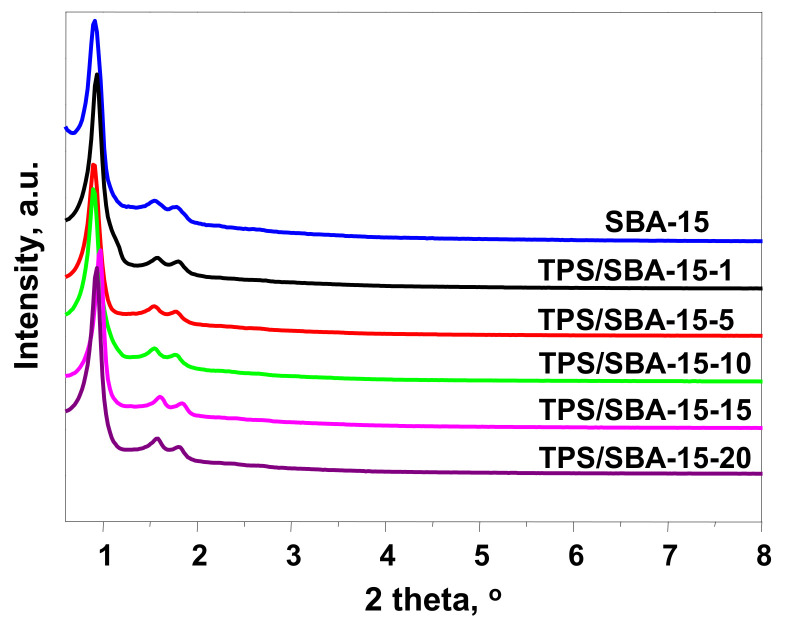
XRD patterns of materials obtained.

The hydrothermal synthesis of mesoporous silica was successful, as confirmed by the XRD patterns and N_2_ ads/des isotherms; a typical XRD pattern of SBA-15 is shown in Figure 1. The intense peak (100) corresponding to the reflections of the hexagonal plane, p6 mm group, is centered at 2 theta of 0.92°. There are two well-resolved additional peaks at 2 theta of 1.55° and 1.77°, related to (110) and (200), respectively. These peaks are not always observed for hexagonal mesoporous silicas. Their appearance means that the hexagonal channels are arranged in the same direction; thus, the sample shows long-range ordering. The use of different pressures of TPS species immobilization did not have a significant impact on the XRD patterns of the materials obtained (Figure 1). For all samples, the (100), (110) and (200) peaks are well resolved, which implies that the modification, even at a pressure of 20 bar, does not have a negative impact on the mesoporous structure and degree of ordering. Nevertheless, for TPS/SBA-15-1, TPS/SBA-15-15 and TPS/SBA-15-20 the (100) peak is slightly shifted to a higher value of 2 theta. It should be mentioned that the position of the (100) peak is related to the regular size distance between the walls of mesoporous channels. Thus, the small shift of the (100) peak position suggests a small decrease in the pore size of the modified material. This feature is commonly observed after post-synthesis modification of mesoporous solids with bulky molecules.

**Figure 2:**
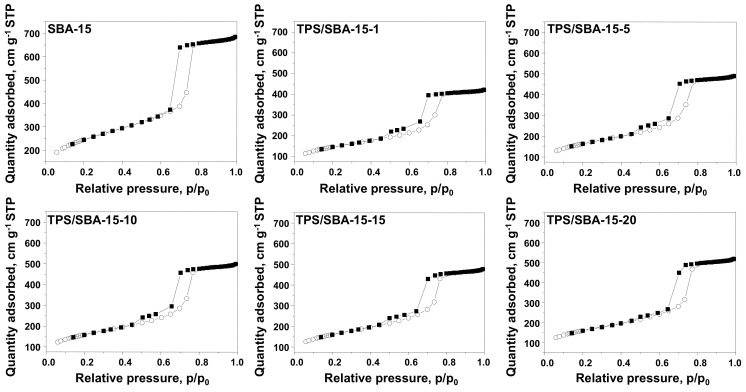
N_2_ adsorption(○)/desorption(■) isotherms of SBA-15 and TPS-containing materials.

The samples obtained were characterized by low temperature N_2_ adsorption/desorption. The resulting isotherms are presented in Figure 2, and the parameters calculated on their basis are provided in Table 1. SBA-15 support shows a typical N_2_ adsorption/desorption isotherm characteristic of this kind of mesoporous material. According to IUPAC classification, the isotherm observed should be assigned as type IVa [16]. The main feature of this isotherm is the presence of a saturation plateau as well as a hysteresis loop. The latter is caused by the condensation of the adsorbate inside the material pores. The presence of narrow and uniform pores is reflected by the H1 type hysteresis loop, which is in line with the high quality XRD pattern recorded for this material. After immobilization of TPS species, the type of isotherm did not change; however, N_2_ uptake decreased for all samples. It should be pointed out that apart from the TPS/SBA-15-20 sample, all modified materials showed the hysteresis loop of type H5. This suggests the presence of both open and partially blocked mesopores [16]. Interestingly, the part of the hysteresis loop at lower values of p/p_0_ seems to decrease with increasing pressure applied during support modification. This suggests that the increase in pressure prevents the pore blockage, as demonstrated by the H1 hysteresis loop observed for TPS/SBA-15-20.

All of the materials obtained had a relatively large surface area, as presented in Table 1. The highest value of surface area was observed for the support, at, 887 m^2^ g^−^^1^. After immobilization of the TPS species, this parameter decreased, ranging from 531 to 594 m^2^ g^−^^1^. Some decrease was also observed in the area of micropores localized in the material walls. These pores are formed upon the calcination of the support after hydrothermal synthesis, i.e., during the thermal removal of Pluronic P123, which is partially occluded by the silica walls. The incorporation of TPS species also affected the pore volume, which for the support reached 0.98 cm^3^ g^−^^1^ and decreased by as much as 0.62 cm^3^ g^−^^1^ for TPS/SBA-15-1. Interestingly, the largest pore volume was observed for the TPS/SBA-15-10 sample. This could be explained by a small wall thickness, which is the same as for TPS/SBA-15-20. The latter material also had a relatively large pore volume. The pore diameter was calculated by the DFT method, using the model for cylindrical pores. This method is preferred for mesoporous materials having a pore diameter of ca. 10 nm or smaller, as the BJH method underestimates this parameter. Interestingly, the pore diameter as estimated by DFT method did not change after incorporation of TPS species. Nevertheless, the pore size distribution estimated from the desorption branch of the isotherm indicates that after immobilization of TPS a dual-pore system is formed (Figures S1 and S2). Except for TPS/SBA-15-10, the position of the main maximum desorption peak of TPS modified samples was almost the same as for the support. However, for all materials, a less intense adsorption maximum was also observed. It seems that TPS is not incorporated into all channels of SBA-15. The pores that are not modified with TPS still have the same diameter, whereas the diameter of the pores modified with TPS decreases. Interestingly, for TPS/SBA-15-10 both peaks exhibit lower pore size values, which is in line with the higher TPS loading shown below.

The XRD patterns and low temperature N_2_ adsorption/desorption isotherms of the reference samples, i.e., Nb/SBA-15 and V/SBA-15, are presented in Figures S3 and S4, while the parameters calculated from the physisorption measurements are presented in Table 1. Briefly, it can be summarized that the incorporation of both modifiers did not have a negative impact on SBA-15 structure. For Nb/SBA-15, no crystalline niobium(V) oxide phase was detected, in contrast to V/SBA-15 material the XRD pattern of which indicated the presence of some V_2_O_5_ phase. The increasing amount of both incorporated metals resulted in a decrease in both the total surface area and the area of micropores. A slight decrease in pore diameter was also observed, especially for Nb/SBA-15.

### 3.2. Efficiency of TPS Incorporation

The generation of acidic centers in mesoporous silica using TPS species as organosilane modifiers ensures that all immobilized species could be considered as Brønsted acid sites. The presence of SO_3_H species has been previously confirmed using FTIR measurements for SBA-15 materials modified with TPS species, where the band at 1380 cm^−^^1^ characteristic of SO_3_H grouping was observed [14]. Since all TPS species contain an SO_3_H grouping, the efficiency of immobilization, and therefore the number of SO_3_H species, can be estimated on the basis of elemental analysis. The results of these measurements are presented in Table 2.

First, it should be mentioned that the SBA-15 support already contains some residual carbon species, which remain after calcination of the material prepared by the hydrothermal one-pot synthesis. The information about residual carbon is especially important if the final material is dedicated for catalytic oxidation processes. It has been documented in the literature that different mesoporous silicas can show some activity in oxidation processes, and that this is related to different radical species, including those coming from carbon residue [17]. As shown in Table 2, after the incorporation of TPS species the amount of carbon is higher than estimated from the C/S ratio in TPS species. Moreover, it should be noted that the difference between the total amount of carbon measured by elemental analysis and the amount of carbon that corresponds to the stoichiometric composition is not the same for all materials. It seems that during modification with TPS species, some residual carbon from the support can be removed, and its amount is not the same in each modification process. Interestingly TPS/SBA-15-10 contained the highest amount of carbon coming from the reagents used in the synthesis of SBA-15.

**Figure 3:**
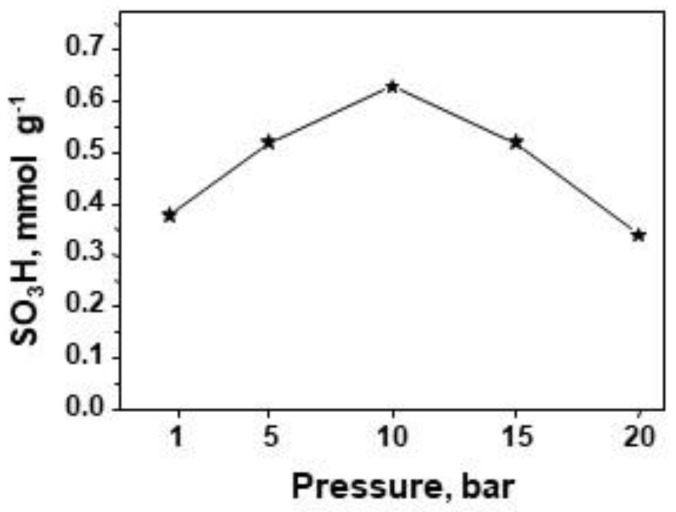
Influence of pressure applied during modification on concentration of acid sites.

One of the objectives of this study was to investigate the impact of pressure on the efficiency of TPS immobilization. The results obtained are presented in Figure 3. The increase in pressure from the ambient value to 10 bar leads to an increase in the concentration of sulfonic species from 0.38 mmol S g^−^^1^ to 0.63 mmol S g^−^^1^. These results are in line with those of previous experiments related to immobilization of (3-mercaptopropyl)trimethoxysilane (MPTMS) on SBA-15, showing that the application of 8.5 bar (the highest pressure used for modification in the relevant work) achieved the highest efficiency of MPTMS incorporation, i.e., 0.62 mmol S g^−^^1^ [13]. Nevertheless, the oxidation of SH species to SO_3_H species in the MPTMS anchored to the support led to partial removal of MPTMS species; the final concentration of sulfur was 0.42 mmolg^−^^1^. In this study the application of pressure from 10 up to 20 bar caused a progressive decrease in TPS species content in the sample. On the basis of the results obtained, it can be concluded that the application of pressure during the incorporation of organosilane species into mesoporous silica enhances the efficiency up to a certain value of pressure, estimated as ca. 10 bar.

### 3.3. Testing of Catalytic Activity and Stability

The presented results allowed determination of the optimal pressure for the immobilization of TPS species on SBA-15 mesoporous silica. The highest efficiency of TPS species incorporation was reached for TPS/SBA-15-10, i.e., for the sample modified under pressure of 10 bar. Therefore, this sample was chosen for the measurement of activity and stability in both the liquid-phase and gas-phase test reactions, i.e., esterification of acetic acid and oxidative dehydration of glycerol, respectively.

#### 3.3.1. Esterification of Acetic Acid with n-Butanol and n-Hexanol

**Figure 4:**
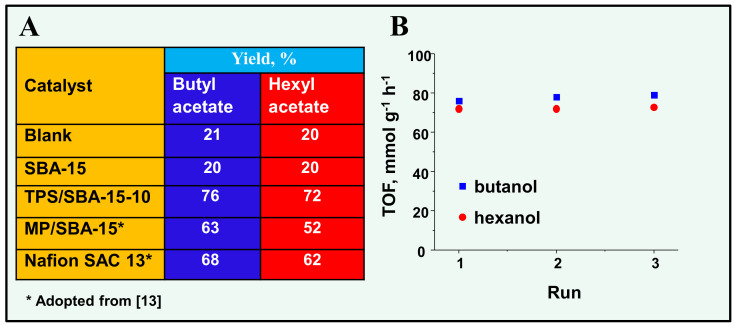
(**A**) Activity in the esterification of acetic acid with n-butanol and n-hexanol; (**B**) Stability and TOF (mmol of acetic acid reacted on 1 g of catalyst during 1 h) of TPS/SBA-15-10 in esterification processes.

Esterification of acetic acid with n-butanol and n-hexanol was chosen as a test reaction in order to check the activity and stability of the optimized material in the liquid phase process. The results obtained are presented in Figure 4.

As acetic acid possesses a mobile proton, the reaction can be autocatalyzed and the final products, i.e., butyl acetate and hexyl acetate, can be observed even for the processes without catalyst addition. The yield of both products was quite significant, reaching ca. 20% (Figure 4A). The addition of a support to the reaction mixture did not influence the results of the process, and the same yield was reached as for the blank test. In the presence of TPS/SBA-15-10, the yield of butyl acetate and hexyl acetate significantly increased, reaching 76% and 72%, respectively. It can be noticed that the catalytic activity (conversion of acetic acid) of the materials obtained within this study was much better than that of the commercial reference catalyst, i.e., Nafion SAC 13, and the catalyst obtained by MPTMS (MP) incorporation (Figure 4). The stability of the TPS/SBA-10 sample is presented in Figure 4B. The presented activity of the catalyst is expressed as TOF values, with the amount of acetic acid reacted in autocatalyzed process (blank test) not included in the calculation. For both processes, the catalytic stability of TPS/SBA-15-10 was evident. It should be mentioned that this result is in line with the previous data reported in the literature for TPS containing mesoporous silica [14].

#### 3.3.2. Oxidative Dehydration of Glycerol

The process of obtaining acrylic acid by oxidative dehydration of glycerol requires selective oxidation of acrolein, formed by glycerol dehydration. The V/SBA-15 catalyst applied in this study did not allow selective glycerol oxidation to acrylic acid; however, other oxidation products which have been previously reported in the literature in the presence of vanadium containing catalysts [18] were detected, i.e., acetic acid and acetaldehyde. Despite the lack of selectivity to acrylic acid, the catalytic tests performed allowed estimation of the activity and stability of TPS/SBA-15-10 in the first step of this process, i.e., dehydration of glycerol.

**Figure 5:**
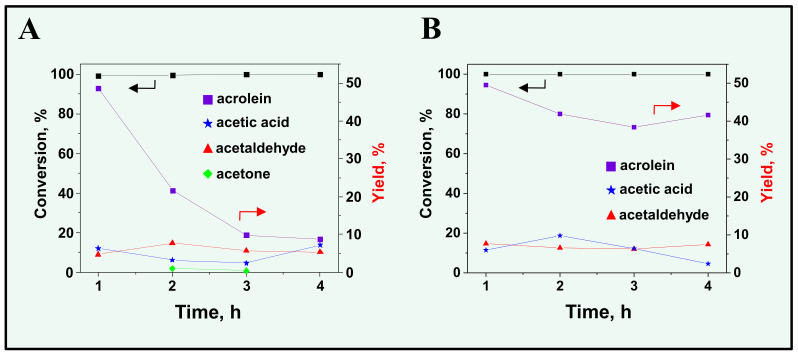
Conversion of glycerol and yield of acrolein in the oxidative dehydration of glycerol on a dual catalytic system: (**A**) TPS/SBA-15-10 + V/SBA-15; (**B**) Nb/SBA-15 + V/SBA-15.

The catalytic activity of the TPS/SBA-15-10 + V/SBA-15 dual system is illustrated in Figure 5A. As mentioned, a total conversion of glycerol was achieved from the beginning of the process. The selectivity to acrolein was ca. 49% in the first hour on stream; however, in the next hour the selectivity decreased to ca. 20%, then to 9% after 4 h. After the reaction, the color of the TPS/SBA-15-10 material changed from white to gray, as presented in Figure S5. It has been previously reported that in the presence of Brønsted acid sites, a decrease in acrolein yield is observed due to coke formation [19,20]. Indeed, the results of the elemental analysis showed that the amount of carbon on the catalyst surface increased from 3.83 mmol g^−^^1^ before the reaction to 9.47 mmol g^−^^1^ after the reaction. Nevertheless, the same analysis showed that there was no sulphur detected after the reaction on the catalyst surface. It should be noted that the conversion of glycerol did not decrease in the time on stream, despite the reduced acrolein yield. At the same time, the yields of the other products detected were rather stable. Therefore, the loss of sulphonic species from the catalyst surface should be the reason for the decrease in selectivity to acrolein. As significant decrease in acrolein selectivity is not accompanied by an increase in the production other products (shown in Figure 5), this suggests that oxidation to CO_2_ occurred.

For comparison, analogous tests were performed for a different catalytic system consisting of Nb/SBA-15 and V/SBA-15. In this system, Nb/SBA-15 has acidic centers which are not as strong as those in TPS/SBA-15-10. It has been previously shown that the incorporation of niobium species on the silica surface generates moderate-strength Lewis acid sites and some Brønsted acidity, although much lower than in H-Al-Si materials [21]. The results obtained are presented in Figure 5B. The initial selectivity to acrolein was ca. 50%; however, in contrast to the TPS/SBA-15-10 + V/SBA-15 material, selectivity did not change as much, reaching ca. 42% after 4 h on stream. It should be mentioned that the color of the Nb/SBA-15 catalyst changed from white to almost black after the reaction (Figure S5). This change in color was accompanied by an increase in the amount of carbon on the material’s surface as measured by elemental analysis (20.33 mmol of C per 1 g of catalyst after the reaction). It should also be noted that despite coke formation on Nb/SBA-15 in much higher amounts than for TPS/SBA-15-10, the catalyst retained its activity and selectivity to acrolein. This allows for the conclusion that the gradual loss of selectivity to acrolein observed for the TPS/SBA-15-10 material is related only to the instability of the organosilane modifier in the reaction condition.

## 4. Conclusions

Post-synthesis modification of SBA-15 surface with 3-(trihydroxysilyl)-1-propanesufonicacid (TPS) was performed under different pressures of up to 20 bar. The increase in pressure during the modification led to an increase in the efficiency of TPS immobilization up to the pressure of 10 bar. Further increases in pressure caused a decrease in the number of modifier molecules on the surface of the SBA-15. Detailed characterization of the samples obtained did not show a negative impact from the pressure applied during modification on the final material structure. The sample obtained in the optimized conditions showed good activity and stability in the esterification of acetic acid with n-butanol and n-hexanol. Nevertheless, TPS/SBA-15-10 was not stable in the dehydration of glycerol in the presence of oxygen in gas-phase, due to a loss of sulphonic species from the catalyst surface. The methodology of the catalyst preparation presented in this study could also be adopted for the modification of silicas with different organosilane species.

## Figures and Tables

**Table 1:** Texture/structure characterization and vanadium incorporation efficiency.

Catalyst	S_Total_^1^ (m^2^ g^−1^)	Pore Diameter ^2^ (nm)	Maximum on PSD Ads ^3^ (nm)	Maxima on PSD Des ^4^ (nm)	S_micro_^5^ (m^2^ g^−1^)	V_Total_^6^ (cm^3^ g^−1^)	Wall Thickness ^7^ (nm)
SBA-15	887	10.6	9.5	7.3	165	0.98	1.30
TPS/SBA-15-1	531	10.6	9.4	7.5; 4.7	104	0.62	1.94
TPS/SBA-15-5	594	10.6	9.5	7.3; 4.7	105	0.73	1.73
TPS/SBA-15-10	573	10.6	8.5	6.5; 3.9	81	0.81	1.58
TPS/SBA-15-15	579	10.6	9.4	7.3; 4.7	90	0.71	1.66
TPS/SBA-15-20	578	10.6	9.4	7.3; 4.7	94	0.78	1.58
Nb/SBA-15	410	9.6	8.3	6.5	33	0.62	1.88
V/SBA-15	438	10.1	8.6	6.4	27	0.67	1.81

^1^ Total surface area as calculated using the BET method. ^2^ pore diameter—calculated using the DFT method. ^3^ PSD—pore size distribution, calculated from the adsorption branch of the isotherm (BJH-KJS method). ^4^ PSD—pore size distribution calculated from the desorption branch of the isotherm (BJH-KJS method). ^5^ Surface area of micropores—t-Plot method. ^6^ Total pore volume—BJH adsorption cumulative volume of pores. ^7^ Wall thickness (t)—t = a_0_ — w/1.05; a_0_ = 1.154700538 × d_100_; w = 1.212522325 × d_100_ × ((δ × V)/(1 + δ × V))^0.5^.

**Table 2:** Results of elemental analysis and concentration of SO_3_H species.

Catalyst	C (mmol g^−1^)	C ^1^ (mmol g^−1^)	S (mmol g^−1^)	C-C ^1^ (mmol g^−1^)
TPS/SBA-15-1	2.33	1.14	0.38	1.19
TPS/SBA-15-5	2.75	1.56	0.52	1.19
TPS/SBA-15-10	3.83	1.89	0.63	1.94
TPS/SBA-15-15	2.78	1.56	0.52	1.22
TPS/SBA-15-20	2.5	1.02	0.34	1.48

^1^ amount of carbon calculated according to C/S molar ratio in TPS species.

## Data Availability

The data presented in this study are available on request from the corresponding author via e-mail: tmaciej@amu.edu.pl (M.T.).

## Supplementary Materials

The following are available online at https://www.mdpi.com/article/10.3390/ma14237226/s1. Figure S1: Pore size distribution estimated from (A) adsorption and (B) desorption branches of N_2_ adsorption/desorption isotherms of SBA-15, TPS/SBA-15-1, TPS/SBA-15-5, TPS/SBA-15-1 materials; Figure S2: Pore size distribution estimated from (A) adsorption and (B) desorption branches of N_2_ adsorption/desorption isotherms of SBA-15-15, TPS/SBA-15-20, Nb/SBA-15, V/SBA-15 materials; Figure S3: XRD patterns of SBA-15, Nb/SBA-15 and V/SBA-15 samples; Figure S4: N_2_ adsorption/desorption isotherms of SBA-15, Nb/SBA-15 and V/SBA-15 samples; Figure S5: Photos of TPS/SBA-15-10 and Nb/SBA-15 catalysts before (A) and after (B) glycerol dehydration in the presence of oxygen.
