# Supplementary material for: Towards Efficient Acidic Catalysts via Optimization of SO3H-Organosilane Immobilization on SBA-15 under Increased Pressure: Potential Applications in Gas and Liquid Phase Reactions
Source: Materials (Basel). 2021 Nov 26;14(23):7226. doi: 10.3390/ma14237226 (PMC8658319; doi:10.3390/ma14237226)
Supplement: Supplementary file 1 [file materials-14-07226-s001.zip › materials-1462420-supplementary.pdf]

Supplementary material

Towards Efficient Acidic Catalysts via Optimization of SO_3H-Organosilane Immobilization on SBA-15 under Increased Pressure: Potential Applications in Gas and Liquid Phase Reactions

Maciej Trejda *, Ada Kaszuba, Ardian Nurwita and Maria Ziolek

Department of Heterogeneous Catalysis, Faculty of Chemistry, Adam Mickiewicz University, Uniwersytetu Poznańskiego 8, 61-614 Poznań, Poland; ada.jadrzak@wp.eu (A.K.); ardnur@st.amu.edu.pl (A.N.); ziolek@amu.edu.pl (M.Z.)

* Correspondence: tmaciej@amu.edu.pl

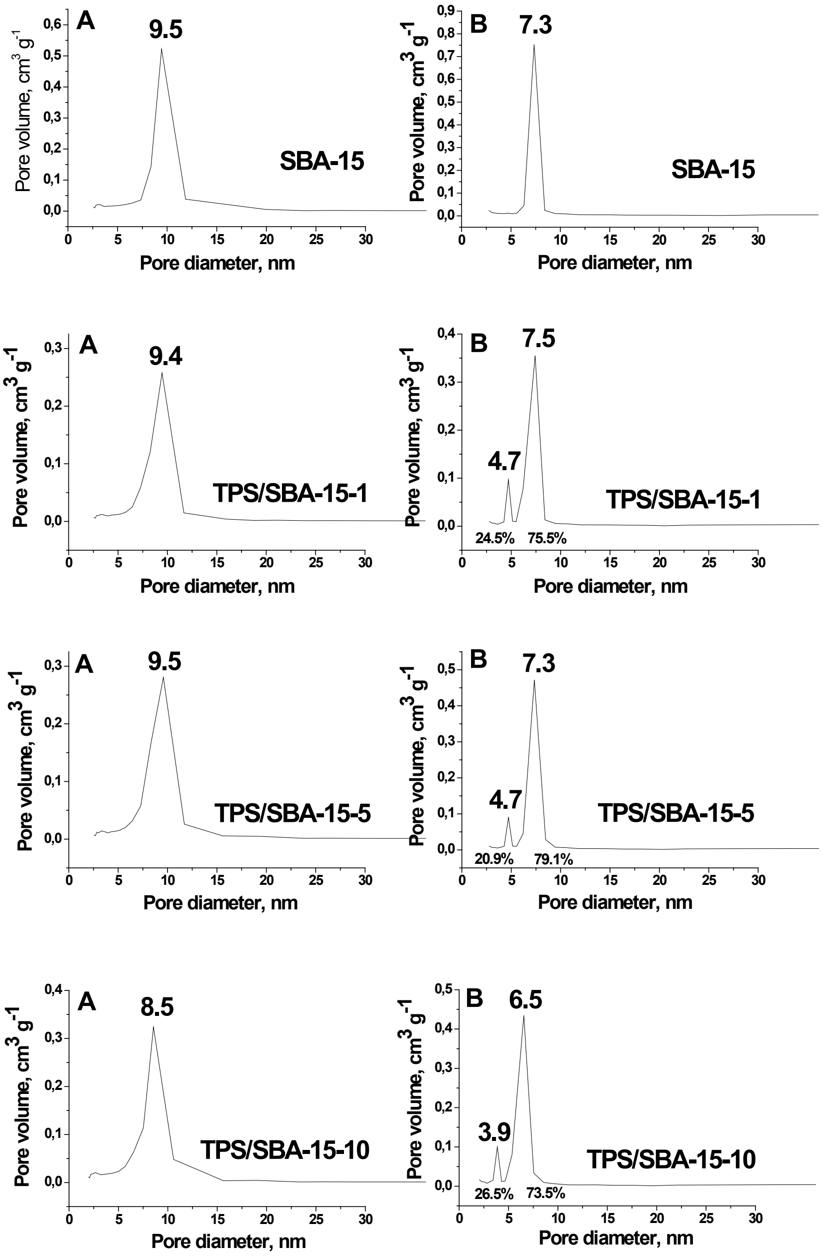

Figure S1. Pore size distribution estimated from (A) adsorption and (B) desorption branches of N_2 adsorption/desorption isotherms of SBA-15, TPS/SBA-15-1, TPS/SBA-15-5, TPS/SBA-15-1 materials.

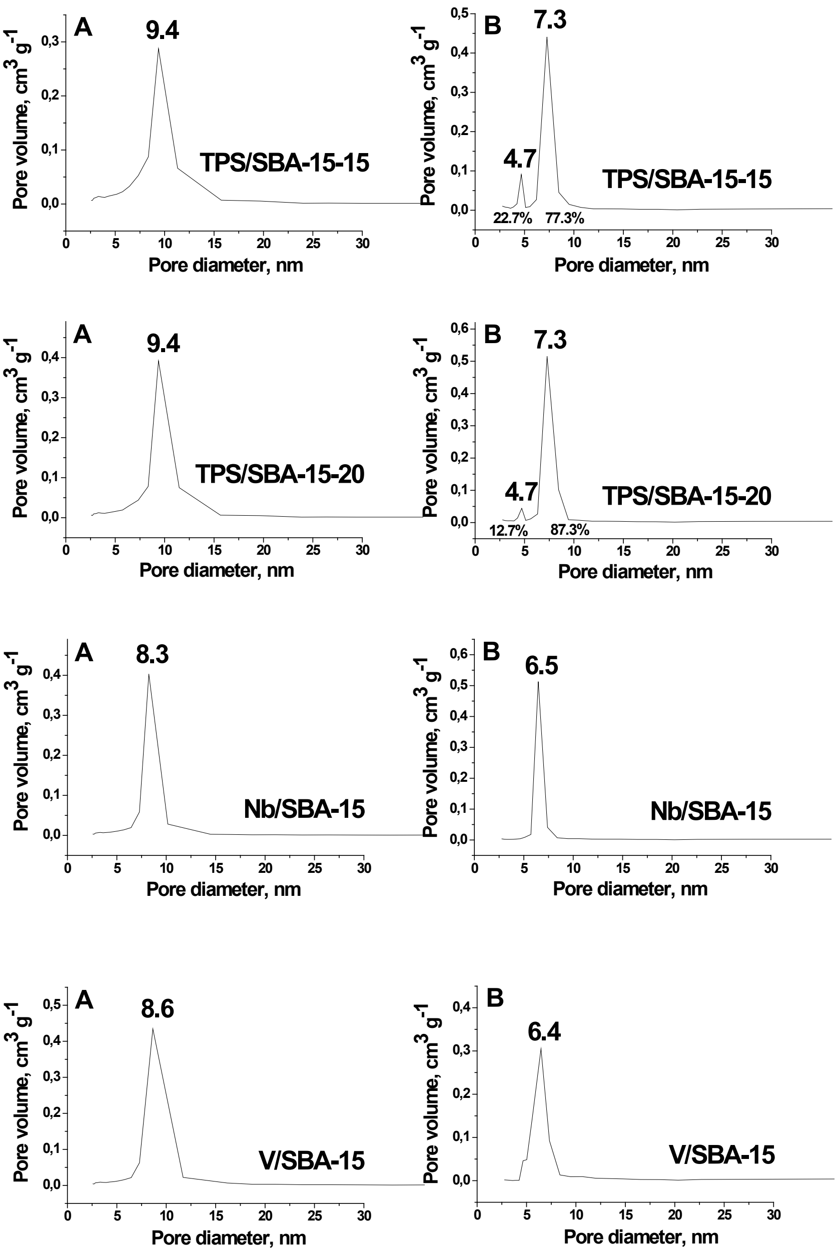

Figure 2. Pore size distribution estimated from (A) adsorption and (B) desorption branches of N_2 adsorption/desorption isotherms of SBA-15-15, TPS/SBA-15-20, Nb/SBA-15, V/SBA-15 materials.

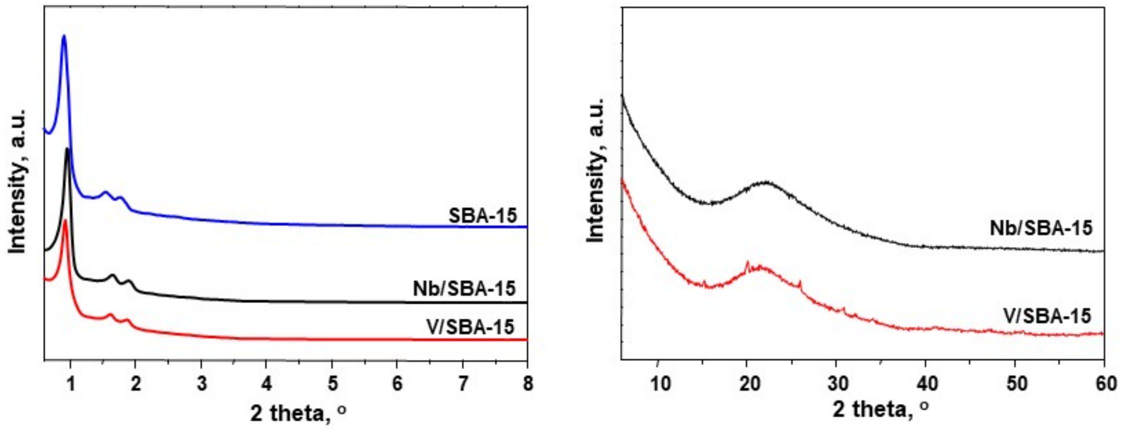

Figure S3. XRD patterns of SBA-15, Nb/SBA-15 and V/SBA-15 samples.

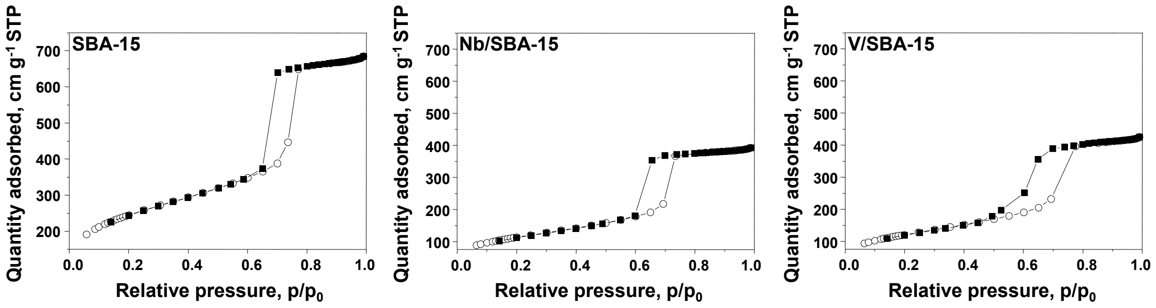

Figure S4. N_2 adsorption/desorption isotherms of SBA-15, Nb/SBA-15 and V/SBA-15 samples.

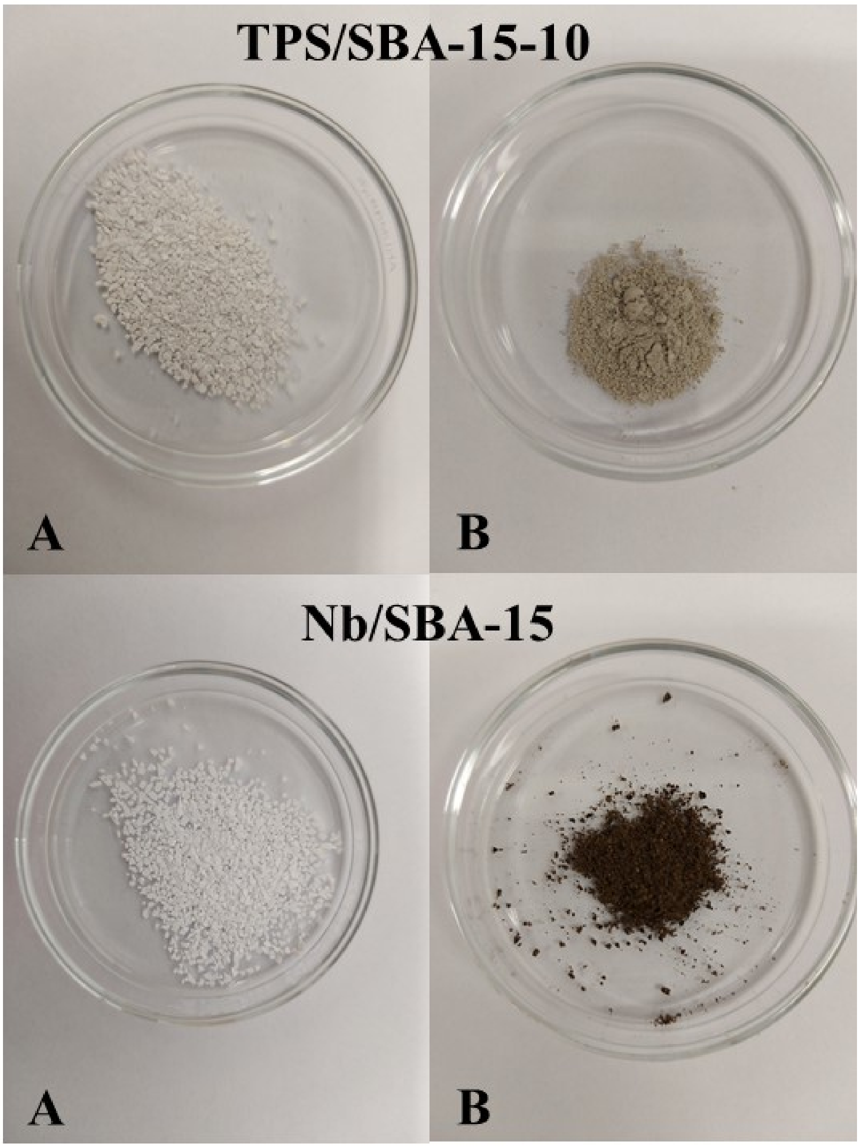

Figure S5. Photos of TPS/SBA-15-10 and Nb/SBA-15 catalysts before (A) and after (B) glycerol dehydration in the presence of oxygen.
